# The impact of employee compensation restrictions on labor productivity in state-owned enterprises: Evidence from China

**DOI:** 10.3389/fpsyg.2022.956523

**Published:** 2022-09-06

**Authors:** Bao Zhu, Zhong Ma, Xiaojie Qu

**Affiliations:** ^1^School of Economics and Management, Beijing Jiaotong University, Beijing, China; ^2^School of Accounting and Auditing, Nanjing Audit University Jinshen College, Nanjing, China

**Keywords:** public policy, employee compensation restriction, labor productivity, behavioral economics, SOEs

## Abstract

Employees are important stakeholders in an organization. This paper aims to examine the effectiveness of limits on employee compensation in state-owned enterprises (SOEs), a policy for employees of state-owned enterprises issued by the China State-owned Assets Supervision and Administration Commission (SASAC) in 2010. Employing a difference-in-differences analysis for a sample of Chinese listed companies from 2007 to 2013, the results show that employee compensation restriction enhances the labor productivity of SOEs. This policy effect is mainly due to the contribution of compensation limits to the external fairness of employee compensation, and the findings remain unchanged after a series of robustness testing procedures. In addition, the employee compensation restriction policy significantly affects labor productivity improvement in monopolistic industries or mature SOEs.

## Introduction

Optimizing wages is one of the main ways to motivate employees in organizations. From 1985 to 2010, China’s state-owned enterprises (SOEs) used a “performance-linked” employee wage management system that led to a rapid increase in employee compensation, along with the development of SOEs (Zhu and Dowling, 1998). However, the inherent flaws of the previous system became increasingly evident following the global financial crisis of 2008, as the salaries of employees in SOEs were much higher than the market average due to factors such as policy resource bias and insider control (Zhou, 2004). In particular, the average wages of SOE employees in monopolistic industries such as electricity and tobacco were two to three times higher than those in other industries. The excessive salary of SOE employees was considered to violate the principle of common prosperity, and is one of the main reasons for the widening income gap in China that is increasingly attracting the attention of regulators, the public, and academics.

This study investigates the effect of a new compensation restriction policy for employees of SOEs. To restrict the compensation of SOE employees, the State-owned Assets Supervision and Administration Commission (SASAC) issued the “Interim Administrative Procedures for the Total Wage Budget of Central State-owned Enterprises” in 2010, followed by the establishment of corresponding systems by the local SASAC. The policy emphasized the establishment of a market-based mechanism for determining the salaries of SOE employees through budgetary management to curb the excess income of SOE employees. Whether employee compensation restrictions promoted efficiency changes in SOEs is a question to be considered. There have been many studies on the incentive effect of salary on employees focusing on salary levels, the salary gap, the minimum wage policy, and employee stock ownership (Holzer, 1990; Beatty, 1995; Dube et al., 2010; Babenko et al., 2011; Xi and Moser, 2011; Fang et al., 2015; Autor et al., 2016; Gan et al., 2016; Hilscher et al., 2021). However, the way employee compensation limits affect labor productivity in SOEs remains unknown.

There is a theoretical ambiguity in the effect of employee compensation restriction on labor productivity in SOEs. On one hand, employee compensation restriction mainly limits the wage level of SOE employees, and according to efficiency wage theory, a cut in average wages may lead to a reduction in employee effort (Shapiro and Stiglitz, 1984; Hannan, 2005); that is, employee compensation restriction may reduce labor productivity in SOEs. On the other hand, individuals usually have decision equity preferences, avoid advantageous unfair outcomes, and are particularly averse to disadvantageous unfair outcomes (Bechtel et al., 2018; Gao et al., 2018; Li et al., 2018). Furthermore, Chinese people pay more attention to the fairness of earnings distribution compared to foreigners (Pillai et al., 2001; Kim and Leung, 2007). Employee compensation limits contribute to higher external equity of employee pay in SOEs and according to social equity theory, increased pay equity can cut down on negative performance behavior such as employee go-slow. Employee compensation limits may also increase labor productivity in SOEs. We look at the empirical question of whether employee compensation restriction has increased labor productivity in SOEs, and attempt to provide systematic evidence for this unexplored issue.

This paper empirically examines the impact of employee compensation restriction on labor productivity of SOEs using the policy restricting employee compensation in 2010 as a quasi-natural experiment. We constructed a difference-in-differences (DID) analysis using the listed firms from 2007 to 2013 and found that: (1) Employee compensation restriction enhances labor productivity of SOEs and the findings remain unchanged after a series of robustness testing procedures. (2) This policy effect is mainly due to the contribution of employee compensation restriction to the external fairness of employee compensation. (3) Employee compensation restriction has a more significant effect on labor productivity improvement in monopolistic industries or mature SOEs.

The contributions of this paper are as follows: First, the findings of this paper suggest that employee compensation restriction policies can effectively balance the “efficiency” and “equity” issues of the initial distribution, thus enriching the relevant theories of policy actions for common prosperity. Previous literature has mainly investigated the effects of equity promotion policies such as executive pay regulation (Dittmann et al., 2011; Ibrahim et al., 2021) and minimum wage policies (Cengiz et al., 2019) on the efficiency of enterprises, ignoring the policy effects of employee compensation restriction of SOEs. Our findings deepen the understanding of employee incentives.

Second, this paper focuses on determining whether employee compensation restrictions can effectively target traditional SOE incentive problems for correction. The study shows that the employee compensation restriction policy can promote external equity in employee compensation, enriching the study of the drivers of SOE efficiency. While the existing literature focuses on the role of corporate governance environment optimization, such as shareholder governance, executive governance, and external governance in enhancing SOE efficiency (Aivazian et al., 2005; Rousseau and Xiao, 2008; Klenow, 2009; Conyon and He, 2011; Liao et al., 2014; Peng et al., 2021), this paper further explores the role of employee compensation restriction in SOE efficiency enhancement, thus providing a useful extension to the literature on the factors influencing SOE efficiency.

## Institutional background

The employee compensation restriction of state-owned enterprises in China has gone through four stages: the stage of the planned economy before 1978; the stage of reform and exploration from 1978 to 1985; the stage of performance-linked pay from 1985 to 2010; and the stage of total wage budget from 2010 to the present.

Before 1978, the egalitarian distribution mechanism was implemented and the wages of employees in state-owned enterprises was decided by the state’s directive plan. State-owned enterprises did not have the autonomy to allocate wages, which frustrated employees’ labor motivation and undermined enterprise productivity (Yue, 1985).

From 1978 to 1985, strict control over the wages of employees in state-owned enterprises was gradually relaxed, and the enterprises made useful attempts to optimize the wage management mechanism through reforms such as re-establishing the principle of distribution according to labor and restoring the piecework wage system.

In 1985 SOEs began to implement a performance-linked employee wage management system (Zhu and Dowling, 1998; Warner, 2010). State-owned enterprises could decide their wage levels and internal distribution methods on the premise of the two lower principles (the growth rate of total wages of employees is lower than the growth rate of economic efficiency of the enterprise, and the growth rate of employee average wages is lower than the growth rate of labor productivity of the enterprise). Since the establishment of the SASAC in 2003, SOEs have had higher autonomy in deciding salaries and this has led to excessive salaries of executives and employees in some enterprises, causing widespread concern.

In 2010, SASAC issued the “Interim Administrative Procedures for the Total Wage Budget of Central State-owned Enterprises.” The total wage budget is a way to manage the total wages of state-owned enterprises, and the SASAC set the wage increase control line for SOEs based on the wage level of the industry to which they belong (Wang, 2014). Local SASACs established the system of total wage budget management for the state-owned enterprises under their jurisdiction, and the total wage management system of state-owned enterprises officially changed from the performance-linked stage to that of total wage budget management.

The policy has changed the assessment indexes and management mode. First, the assessment index system has changed from being linked to the company’s performance to being double-linked to the company’s performance and market-oriented wage level, and the wage increase control line incorporated the average social wage, labor market price, and other market-oriented wage indicators. Second, the policy changed the management mode: in the performance-linked stage, the SASAC had approved the total wages of SOEs at the end of the year, while in the total wage budget management stage, the SASAC adopts the management mode of planning at the beginning of the year, implementing and monitoring in the middle of the year, and liquidating at the end of the year. In this way the SASAC has more control over the wages of SOEs’ employees.

## Hypothesis development

As important internal stakeholders, employees are an essential subject of enterprise value creation, and wages are a means to motivate employees to improve their work enthusiasm (Schultz, 1961; Shapiro and Stiglitz, 1984). The compensation restriction policy emphasized the linkage between employee wage increases and enterprise performance while considering the adaptation of wage levels to the labor market. Compensation fairness affects employee salary incentives; the marketization of the employee wage determination mechanism may change the external fairness of employees’ pay, which in turn affects enterprises’ labor productivity.

Social equity theory suggests that employee satisfaction with income affects employee motivation, and due to an aversion to individual unfairness, employee satisfaction with pay depends not only on their absolute pay but also on their relative pay, and relative perceptions of fairness in income affect employee motivation (Adams, 1963). Unfairness aversion encompasses both disadvantageous unfairness and advantageous unfairness, and individuals are averse not only to disadvantageous unfair solutions that are unfavorable to them but also to advantageous unfair solutions that are favorable to them (Yu et al., 2013). Card et al. (2012) found that individuals paid below the industry median showed lower job satisfaction, while those paid above the industry median did not show higher satisfaction. When pay is at disadvantageous inequity, workers will lose avoidance psychology and engage in opportunistic behavior. When pay is at advantageous inequity, workers will lose their future labor motivation due to excessive wage rewards without more effort and may reduce their self-evaluation, leading to the negative tendency of slacking off, maintaining the status quo, and not thinking of progressing. Thus, relatively high external pay is consistent with self-interest preferences but contrary to equity preferences does not bring higher job satisfaction to workers (Camerer, 2003). Therefore, weakening the unfairness of workers’ pay advantage can have a positive effect on workers’ efforts.

From the perspective of external fairness of employees’ remuneration, the policy has strengthened the market determination of employees’ wages and enhanced the external fairness of employees’ remuneration. In the past, the lack of external fairness of employee remuneration in state-owned enterprises was reflected in the fact that employee remuneration was too high relative to their industries or labor markets, and the external remuneration gap was too large. The new policy required the key indicators in the total wage determination mechanism to be scientifically benchmarked with the labor market and the industries to which they belong, and to strengthen the regulation of state-owned enterprises with total wages significantly higher than the industry average. This would strengthen the external fairness of employees’ remuneration. Individuals pay attention to fairness, even at the expense of punishing unfairness (Fehr and Gächter, 2002; Camerer, 2003). By limiting the unreasonable income of employees, employee compensation restriction can, to a certain extent, restrain the phenomenon of “reaping without sowing” and enhance the efforts of employees in SOEs. Thus, by narrowing the gap between employees’ external pay, employee compensation restriction in SOEs enhances employees’ perceived external pay equity, boosts their motivation to work, and contributes to the improvement of company performance and efficiency.

In contrast, from the perspective of efficiency-wage theory (EWT), employee compensation restriction may also have a negative significant effect on labor productivity in SOEs. The efficiency-wage theory suggests that higher wages tend to have a more significant incentive function for workers (Shapiro and Stiglitz, 1984; Leonard, 1987), and higher wages can lead to higher firm performance. For the following reasons, we believe that EWT is not an appropriate perspective to explain the incentive effect of reducing employee pay in SOEs in China. In past practice, the wage system of SOEs established a performance-related pay system, while the labor cost constraints available to SOEs for allocation to employees were low due to factors such as skewed credit policies and market preferences (Brandt and Li, 2003; Gordon and Wei, 2003; Li et al., 2008; Haveman et al., 2017). The pay level of employees in SOEs in China is too high compared to private enterprises, the mobility of employees in state-owned enterprises is poor, and the degree of labor market constraints is low. Previous studies have shown that high wages are due to the excessive welfare of employees in SOEs (Bai et al., 2019; Jian et al., 2020; Kong et al., 2020), an important factor in aggravating the low efficiency of SOEs and is inconsistent with the basic assumptions of the efficiency-wage theory.

H1: Employee compensation restriction increases labor productivity in state-owned enterprises.

H2: Improving the external equity of employee compensation is the main mechanism by which the employee compensation restriction policy improves labor productivity in state-owned enterprises.

There may be industry-level heterogeneity in the effects of employee compensation restrictions on labor productivity in SOEs. A well-developed market competition mechanism can encourage firms to set optimal pay contracts for employees, an effective mechanism to motivate employees (Chung, 2002). The pay levels of SOEs in perfectly competitive industries have already reached the Pareto optimum, and employee compensation restrictions may distort the otherwise effective pay incentives. However, SOEs in monopolistic industries such as electricity and tobacco in China lack the constraints of market mechanisms. With more economic resources and policy preferences, the wages of SOE employees are not sufficiently matched with their abilities, and the proportion of employees’ luck pay is high. Employee compensation restriction can further promote the external fairness of pay for employees in SOEs in monopolistic industries, and thus enhance their efforts. Therefore, employee compensation restriction has a more pronounced effect on the labor productivity enhancement of SOEs in monopolistic industries.

H3: Employee compensation restriction policy has a more significant effect on labor productivity enhancement of monopoly industry SOEs relative to competitive industry firms.

The effect of employee compensation restriction on labor productivity enhancement of SOEs has a life-cycle heterogeneity. Life cycle theory suggests that at different life cycle stages, corporate strategic decisions, investment and financing decisions, and profit distribution decisions have great variability, and employee compensation strategies should be formulated according to life cycle variability (Ellig, 1981). As firms move from growth to maturity, employees are less sensitive to incentives such as promotions, but pay more attention to pay equity, and the promotion of pay equity to firm value is more significant. If promoting external equity in employee compensation is an effective channel for payroll control to enhance labor productivity in SOEs, then it can be expected that the payroll would have a more significant role in enhancing labor productivity in mature SOEs.

H4: Employee compensation restriction policies are more effective in enhancing the labor productivity of mature SOEs relative to mature-period firms.

## Data, variables, and methodology

### Sample and data

To examine the effect of employee compensation restriction on labor productivity of SOEs, this paper uses A-share listed companies in China from 2007 to 2013 as the initial research sample and excludes the following samples: financial industry companies, ST companies, companies with missing data, and pre-pilot companies. After the above processing procedures, we finally obtained 10,943 research samples. Company basic information data were obtained from the Flush database, and other data were obtained from the China Stock Market and Accounting Research Database (CRSMAR). To exclude outliers from perturbing the empirical test results, we winsorized all continuous variables on the 1 and 99% quantiles.

Since the local SASACs followed up the employee compensation restriction policy in 2010, employee compensation in central and local SOEs are both limited, while private enterprises are not affected by the change in this policy. Drawing on existing studies related to the evaluation of compensation control policies (Bai et al., 2019), we use SOEs as the experimental group and private enterprises as the control group in a quasi-natural experiment. A shorter sample period can minimize the interference of other factors unrelated to the pay restriction policy in the empirical setting. To exclude the influence of other exogenous factors on the findings of this paper, we selected 3 years before and 3 years after the policy as the sample period for the benchmark test. The sample interval setting of an equal number of years before and after the policy implementation also ensures the comparability of the samples before and after the policy implementation.

### Model and variables

We estimated Eq. (1) to test the effect of employee compensation controls on labor productivity in SOEs:


$${\text{Labpro}}_{\text{it}}=\alpha +\beta_{1}\,\mathrm{Post}*{\mathrm{SOE}}_{\text{it}}+\beta_{2}\,{\text{Size}}_{\text{it}}+\beta_{3}\,{\text{Lev}}_{\text{it}}$$

$$\;+\beta_{4}\,{\text{Cash}}_{\text{it}}+\beta_{5}\,{\text{Growth}}_{\text{it}}+\beta_{6}\,{\text{ROE}}_{\text{it}}$$

$$\;+\beta_{7}\,{\text{Dual}}_{\text{it}}+\beta_{8}\,{\text{Conc}}_{\text{it}}+\beta_{9}\,{\text{Direct}}_{\text{it}}$$

(1) $$\;+\beta_{10}\,{\text{Inde}}_{\text{it}}+\beta_{11}\,{\text{Age}}_{\text{it}}+\mathrm{Year}+\mathrm{Indus}+\mathrm{Prov}+\varepsilon$$

where *Labpro* is labor productivity. Drawing on measures from the existing literature (Bender et al., 2018; Kale et al., 2019), the natural logarithm measure of enterprise unit labor output (operating income/number of employees), and the main explanatory variable in the model is Post*SOE, with *Post* as a dummy variable before and after the policy. Post*SOE is the interaction term between *Post* and *SOE*, and the coefficient of this term is the object of our attention; if significant, it indicates that the causal relationship between employee compensation control and labor productivity is initially established. Consistent with existing studies (Cheng, 2018; Hoang et al., 2021), the model also controls for firm size (*Size*), leverage ratio (*Lev*), the ratio of cash to assets (*Cash*), sales growth ratio (*Growth*), return on asset (*ROA*), CEO duality (*Dum*), the board size (*Board*), independence of the board of directors (*Inde*), concentration of shareholding (*Conc*), and the number of years that the company is listed (*Age*), We also control for the year fixed effects, industry fixed effects, area fixed effects, and cluster standard errors at the firm level. Table 1 provides definitions of all these variables and data sources.

**TABLE 1:** Variable definitions.

Variable	Definition
*Labpro*	The radio of sales to the number of employees
*Post*	A dummy variable equals 1 if the year of the sample is 2010 onwards, 0 otherwise.
*SOE*	A dummy variable equals 1 if the actual controller of the listed company is a state institution, 0 otherwise
*Size*	The natural logarithm of total assets at the end of year t.
*Lev*	Total liabilities divided by total assets at the end of year t.
*Cash*	Cash and cash equivalents divided by total assets at the end of year t.
*ROE*	Net income in year t divided by total assets.
*Growth*	Sales in year t minus sales in year t−1, then divided by sales in year t−1.
*Dual*	A dummy variable equals 1 if the chair of the board and CEO are the same person, 0 otherwise.
*Conc*	The ownership percentage of the largest shareholder.
*Direct*	The number of board directors.
*Inde*	The radio of independent directors to the number of board directors
*Age*	The number of years that the company is listed

## Results

### Descriptive statistics

Table 2 reports the results of descriptive statistics for the main variables. The mean value of labor productivity (*Labpro*) is 11.82 with a standard deviation of 0.983, which is generally consistent with the results of the previous studies (Bao et al., 2014; Cheng, 2018). The dummy variable before and after the policy (*Post*) has a mean value of 0.375, indicating a higher percentage of the sample before the year of policy introduction. For the control variables, the mean value of *Size* is 21.81, the mean value of leverage (*Lev*) is about 47%, the mean value of cash holding ratio (*Cash*) is about 20%, the mean value of corporate growth (Growth) is 0.21, and the mean value of corporate return on net assets (*ROA*) is 0.07. The mean values of *Dual*, *Conc*, and *Inde* are 0.196, 36.85, and 0.368, respectively. This indicates that most of the sample listed companies are characterized by the separation of the chairman and the CEO, the concentration of the shareholding of the largest shareholder, and having about 40% of the board members as independent directors. Overall, the control variable statistics are consistent with existing studies (Chen et al., 2018; Hoang et al., 2021).

**TABLE 2:** Descriptive statistics.

Variable	*N*	Mean	SD	Min	P50	Max
*Labpro*	10,943	13.65	0.983	11.82	13.52	16.57
Post*SOE	10,943	0.325	0.468	0	0	1
*Size*	10,943	21.81	1.259	19.58	21.64	26.09
*Lev*	10,943	0.470	0.219	0.055	0.479	0.944
*Cash*	10,943	0.192	0.142	0.013	0.153	0.621
*Growth*	10,943	0.214	0.492	−0.574	0.136	3.273
*ROE*	10,943	0.070	0.133	−0.709	0.075	0.373
*Dual*	10,943	0.196	0.397	0	0	1
*Conc*	10,943	36.85	15.26	9.274	35.50	74.66
*Direct*	10,943	9.038	1.789	5	9	15
*Inde*	10,943	0.368	0.051	0.308	0.333	0.571
*Age*	10,943	8.907	5.582	0	9	23

### The effect of employee compensation controls on labor productivity

To test the effect of wage control policies on firms’ labor productivity, model (1) is estimated. Table 3 reports the results of testing hypothesis H1, with column (1) showing the results of the OLS (ordinary least square) method controlling for year, industry, region, and individual effects but not including control variables, and column (2) showing the results of the OLS test including all control variables. The results show that the coefficient of Post*SOE is significantly positive for both the inclusion of all control variables and the control of some variables, indicating that the wage bill control policy significantly enhances labor productivity in SOEs. Hypothesis H1 was therefore verified. The direction of the coefficients of the control variables is generally consistent with the results of existing studies (Hoang et al., 2021; Li et al., 2021) with the larger size, higher debt ratio and equity concentration, and higher liquidity and growth being associated with higher labor productivity, while the combination of two jobs is negatively associated with labor productivity of firms.

**TABLE 3:** Employee compensation controls and labor productivity.

	(1)	(2)
	*Labpro*	*Labpro*
Post*SOE	0.2892***	0.0806**
	(8.4163)	(2.1767)
*Size*		0.2286***
		(13.5207)
*Lev*		0.3518***
		(3.4240)
*Cash*		0.3590***
		(2.9916)
*Growth*		0.2045***
		(9.8056)
*ROE*		0.4951***
		(5.5870)
*Dual*		−0.0980***
		(−2.9048)
*Conc*		0.0032***
		(2.8767)
*Direct*		−0.0100
		(−0.9358)
*Inde*		−0.3709
		(−1.2891)
*Age*		0.0000
		(0.0091)
Constant	13.6555***	8.5624***
	(99.2958)	(23.0157)
Year FE	Yes	Yes
Industry FE	Yes	Yes
Province FE	Yes	Yes
N	10,943	10,943
Adj-R2	0.2497	0.3611

T-values are reported in parentheses; ** and *** indicate statistical significance at the 5 and 1% significance level, respectively.

### Robustness tests

#### Parallel trend analysis

The valid estimation of the DID model presupposes that the experimental and control groups satisfy the parallel trend assumption. There are two ways of testing the parallel trend assumption: one is to determine whether the DID model satisfies the parallel trend assumption based on the time trend of the mean values of the dependent variables in the experimental and control groups before and after the policy (Li et al., 2016). The other is to include the interaction term between the dummy variables and the policy variables in each year in the regression and determine whether the DID model satisfies the parallel trend assumption based on the significance of the coefficients of the interaction term (Beck et al., 2010). This paper uses both ideas to test whether the assumptions of the baseline regression model are valid.

**FIGURE 1:**
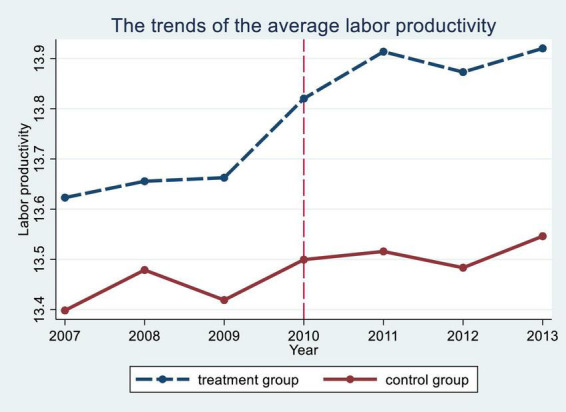
Parallel trends.

Figure 1 shows the annual trends of labor productivity in the experimental and control groups. The results show that the labor productivity of SOEs and private firms maintained approximately the same growth trend before the payroll control policy, but after the payroll control policy was introduced, the labor productivity growth trend of SOEs and private firms changed significantly. Therefore, the graphs of annual labor productivity trends in the experimental and control groups show that the baseline regression model satisfies the assumption of parallel trends.

Table 4 shows the regression results with the interaction term of dummy variables and policy variables for each year, and whether there is a significant difference between the labor productivity of the experimental and control groups in each year before the policy. The results show that the coefficients *dupre1*, *dupre2*, and *dupre3* of the constructed variables in the 3 years before the wage bill policy are not significant, indicating that there is no significant difference between the labor productivity of state-owned enterprises and private enterprises before the issuance of the wage bill policy, and that the pre-policy control group and the treatment group satisfy the parallel trend test. In addition, the coefficients of *dupost1* and *dupost2* are significantly positive at the 5 and 10% levels, respectively, while the coefficient of *dupost3* is insignificant, verifying the problem of insufficient persistence of the effect of the above-mentioned employee payroll control policies to improve labor productivity in state-owned enterprises.

**TABLE 4:** Results of alternative parallel trend tests.

	*Labpro*
*dupre1*	0.0254
	(0.4633)
*dupre2*	−0.0104
	(−0.1809)
*dupre3*	0.1024
	(1.6073)
*current*	0.0919*
	(1.8462)
*dupost1*	0.1114**
	(2.3989)
*dupost2*	0.0814*
	(1.8799)
*dupost3*	0.0580
	(1.3458)
*Size*	0.2279***
	(13.3797)
*Lev*	0.3552***
	(3.4482)
*Cash*	0.3643***
	(3.0132)
*Growth*	0.2047***
	(9.7812)
*ROE*	0.4972***
	(5.6143)
*Dual*	−0.0972***
	(−2.8741)
*Conc*	0.0031***
	(2.8213)
*Direct*	−0.0105
	(−0.9785)
*Inde*	−0.3695
	(−1.2844)
*Age*	−0.0000
	(−0.0109)
Constant	8.5092***
	(22.7267)
Year FE	Yes
Industry FE	Yes
Province FE	Yes
N	10,943
Adj-R2	0.3611

T-values are reported in parentheses; *,** and *** indicate statistical significance at the 1%, 5%, and 10% levels, respectively.

#### Propensity score matching-difference-in-differences

The sources of endogeneity problems are mainly mutual causality, omitted variables, and measurement errors. Employee compensation control is an exogenous shock for enterprises, and although the adoption of the difference-in-differences model (DID) can avoid the endogeneity problems caused by mutual causality, the experimental and control groups in the sample of this paper are state-owned and private enterprises and the systematic differences between the two themselves may be the common cause of labor productivity heterogeneity. The empirical results estimated by the baseline regression model may be subject to endogeneity bias caused by omitted variables.

To alleviate this endogeneity problem, we used the PSM-DID model to reexamine the causal relationship between employee pay controls and labor productivity. The covariates in the propensity score matching (PSM) stage are the control variables in the benchmark regression model, and the matching method is least-nearest-neighbor matching. The estimated results of the PSM-DID model are shown in column (1) of Table 5, and the estimated coefficient of Post*SOE is 0.1038, which is significantly positive at the 5% level of significance and is generally consistent with the results of the benchmark regression.

**TABLE 5:** Other robustness test results.

	(1)	(2)	(3)	(4)
	*Labpro*	*Labpro*	*Labpro3*	*Labpro*
Post*SOE	0.1038**		0.0794**	0.0373**
	(2.2918)		(1.9889)	(2.0267)
Post1*SOE		0.0285*		
		(1.6600)		
*Size*	0.2206***	0.2316***	0.2519***	0.2359***
	(9.3495)	(13.7258)	(13.2261)	(16.9552)
*Lev*	0.4886***	0.3612***	0.2730**	−0.1812***
	(3.2700)	(3.5361)	(2.3283)	(−3.3795)
*Cash*	0.7208***	0.3553***	0.3900***	0.1838***
	(3.6901)	(2.9602)	(2.9649)	(3.0509)
*Growth*	0.1525***	0.2010***	0.2385***	0.2018***
	(4.7912)	(9.6405)	(10.0796)	(21.6559)
*ROE*	0.5660***	0.4882***	0.4460***	0.3166***
	(4.0716)	(5.4800)	(4.1056)	(8.1840)
*Dual*	−0.0660	−0.1069***	−0.1212***	−0.0621***
	(−1.3451)	(−3.1852)	(−3.3333)	(−3.1859)
*Conc*	0.0046***	0.0034***	0.0039***	0.0047***
	(3.1166)	(3.0784)	(3.3017)	(5.1055)
*Direct*	−0.0123	−0.0079	−0.0110	−0.0271***
	(−0.8379)	(−0.7455)	(−0.9850)	(−4.3883)
*Inde*	−0.1052	−0.3748	−0.4433	−0.2902*
	(−0.2715)	(−1.3017)	(−1.4504)	(−1.8063)
*Age*	0.0034	0.0020	−0.0003	0.0266***
	(0.7336)	(0.5954)	(−0.0883)	(6.7203)
Constant	8.5244***	8.4530***	8.1135***	8.4288***
	(17.1735)	(22.9559)	(19.8657)	(29.4955)
Year FE	Yes	Yes	Yes	Yes
Industry FE	Yes	Yes	Yes	No
Province FE	Yes	Yes	Yes	No
Firm FE	No	No	No	Yes
N	4,064	10,943	10,868	10,943
Adj-R2	0.3474	0.3605	0.3459	0.194

T-values are reported in parentheses; *,** and *** indicate statistical significance at the 1%, 5%, and 10% levels, respectively.

#### Placebo test

To exclude possible perturbations in the baseline regression results from other unobserved policies, we re-tested the relationship between employee compensation limits and labor productivity using a placebo test. Specifically, we re-tested by randomly dividing the sample into a control group and an experimental fictitious treatment group. Column (2) of Table 5 reports the results of the placebo test for the fictitious policy treatment group versus the control group. The results show that the coefficient on the policy effect (Post1*SOE) is insignificant, indicating that the effect of wage bill policy on labor productivity improvement in SOEs is unlikely to be result-driven by unobservable factors.

#### Alternative variable

We also measure labor productivity using an alternative variable, which is captured by the natural logarithm of the ratio of the main business income to the number of employees, for which we re-tested using this indicator as an alternative measure of labor productivity. Column (3) of Table 5 reports the results of the test using the replacement explanatory variables, and the results show that the coefficients of Post*SOE are all significantly positive after replacing the measure of labor productivity. The empirical findings that employee compensation controls promote labor productivity in SOEs remain unchanged.

#### Fixed effect model

Labor productivity has a strong individual continuity influenced by individual factors of enterprises. We further used a fixed effect model to control individual effect. Column (4) of Table 5 reports the results of the test using the fixed effect model and the results show that the coefficients of Post*SOE is positive at the 5% level of significance. The empirical findings that employee compensation restriction promotes labor productivity in SOEs remain unchanged.

## Mechanism analysis and cross-sectional analyses

### The mediating role of external fairness in compensation

To test the mediating effect of pay external fairness, the stepwise analytical test model of Baron and Kenny (1987) was used to verify the validity of the above mediating variables (Baron and Kenny, 1987).

Following Zhang et al. (2021), we measured the external fairness of employee compensation by matching the industry ranking of firm performance with the industry ranking of employee compensation, the difference between the profitability ranking of the firm’s industry in each year, and the inverse ranking of average employee compensation (normalized by the number of listed companies in the industry). The higher the indicator, the higher the degree of the unfairness of SOE employee compensation in the current year.

The results of the intermediate effects are shown in Table 6. Column (2) shows the regression results of enterprise labor productivity on policy variables and of external fairness of employee compensation on policy variables. Column (3) shows the regression results of enterprise labor productivity on external fairness of employee compensation with policy variables. The test results show that the coefficient of Post*SOE in column (2) is significantly positive at the 5% significance level, indicating that the external fairness of employee compensation in SOEs is higher after the introduction of the employee compensation control policy. The coefficient of *Equi* in column (3) is significantly positive at a 1% level of significance, indicating that enhancing the external fairness of employee compensation is conducive to enhancing labor productivity in SOEs. The coefficient of Post*SOE is not significant, indicating the full mediation effect of external fairness of employee compensation. The results of the mediation effect test indicate that external fairness of employee compensation is an effective employee compensation control in improving labor productivity in SOEs.

**TABLE 6:** The result of the mechanism analysis.

	(1)	(2)	(3)
	*Labpro*	*Equi*	*Labpro*
Post*SOE	0.0806**	0.1201***	−0.0176
	(2.1767)	(8.3487)	(−0.5085)
*Equi*			0.8169***
			(20.3511)
*Size*	0.2286***	0.0204***	0.2119***
	(13.5207)	(3.6078)	(13.5997)
*Lev*	0.3518***	0.3658***	0.0530
	(3.4240)	(10.1797)	(0.5576)
*Cash*	0.3590***	−0.0087	0.3661***
	(2.9916)	(−0.1956)	(3.3273)
*Growth*	0.2045***	−0.0557***	0.2500***
	(9.8056)	(−6.8960)	(12.3737)
*ROE*	0.4951***	−0.9866***	1.3010***
	(5.5870)	(−17.8145)	(12.3055)
*Dual*	−0.0980***	−0.0226*	−0.0796***
	(−2.9048)	(−1.7904)	(−2.6014)
*Conc*	0.0032***	0.0004	0.0028***
	(2.8767)	(1.1965)	(2.7842)
*Direct*	−0.0100	0.0057*	−0.0147
	(−0.9358)	(1.7272)	(−1.5342)
*Inde*	−0.3709	0.1819*	−0.5195**
	(−1.2891)	(1.7916)	(−1.9953)
*Age*	0.0000	0.0034***	−0.0027
	(0.0091)	(2.7329)	(−0.8546)
Constant	8.5624***	−0.5031***	8.9733***
	(23.0157)	(−4.1649)	(26.3261)
Year FE	Yes	Yes	Yes
Industry FE	Yes	Yes	Yes
Province FE	Yes	Yes	Yes
N	10,943	10,943	10,943
Adj-R2	0.3611	0.2870	0.4341

T-values are reported in parentheses; *,** and *** indicate statistical significance at the 1%, 5%, and 10% levels, respectively.

### Subsamples of monopolistic industries vs. competitive industries

We further test the heterogeneity effects of the industry, industries with the following markings in the broad industry codes were used as monopolistic industries: B06 (coal mining and washing industry), B07 (oil and natural gas mining industry), B08 (ferrous metal mining and beneficiation industry), B09 (non-ferrous metal mining and beneficiation industry), B10 (non-metallic mining and beneficiation industry), B11 (other mining industry), C16 (tobacco products industry), C25 (petroleum processing, coking and nuclear fuel processing industry), C26 (chemical raw materials and chemical products manufacturing industry), C27 (pharmaceutical manufacturing industry), C28 (chemical fiber), C29 (rubber products industry), C32 (ferrous metal smelting and rolling processing industry), C33 (non-ferrous metal smelting), C35 (general equipment manufacturing industry), C36 (special equipment manufacturing industry), C37 (transportation equipment manufacturing industry), C40 (communication equipment, computer and other electronic equipment manufacturing industry), D44 (power and heat production and supply industry), D45 (gas production and supply industry), D46 (water production and supply industry are defined as monopoly), and the rest are competitive industries. The sample is divided into monopolistic industries (Comp = 1) and competitive industries (Comp = 0), and the impact heterogeneity of monopolistic industries and competitive industries is tested by group regression. The results of the grouped regressions are shown in Table 7, with (1) showing the regression results for the monopoly industry sample and (2) showing the regression results for the competitive industry sample. The results show that the coefficient of Post*SOE is significantly positive at the 1% level in the monopoly industry sample and insignificant in the competitive industry sample. This indicates that employee compensation control has a more significant effect on the productivity improvement of SOEs in the monopoly industry, and hypothesis H4 is verified.

**TABLE 7:** Monopolistic industries vs. competitive industries.

	(1)	(2)
	*Labpro*	*Labpro*
Post*SOE	0.1468***	−0.0373
	(3.0988)	(−0.6725)
*Size*	0.2104***	0.2627***
	(9.8597)	(10.0347)
*Lev*	0.5210***	0.1272
	(3.8497)	(0.8705)
*Cash*	0.4124***	0.2688
	(2.6155)	(1.6024)
*Growth*	0.2156***	0.1688***
	(8.4718)	(5.1317)
*ROE*	0.4820***	0.4387***
	(3.6638)	(4.0675)
*Dual*	−0.1108**	−0.0679
	(−2.4587)	(−1.5113)
*Conc*	0.0050***	0.0003
	(3.4539)	(0.1720)
*Direct*	−0.0284**	0.0120
	(−2.0656)	(0.7662)
*Inde*	−0.5411	−0.1926
	(−1.4642)	(−0.4605)
*Age*	−0.0023	0.0029
	(−0.5001)	(0.5561)
Constant	9.0902***	7.5881***
	(19.3929)	(14.5703)
Year FE	Yes	Yes
Industry FE	Yes	Yes
Province FE	Yes	Yes
N	6,827	4,116
Adj-R2	0.4112	0.2794

T-values are reported in parentheses; ** and *** indicate statistical significance at the 5 and 1% significance level, respectively.

### Subsamples of growth stage and maturation stage

To test the heterogeneity of the firm’s life period, we split our sample into subsamples of growth stage and maturation stage and retested. Drawing on Dickinson’s (2011) measure, and considering the listing requirements of companies in China, companies that pass the A-share listing audit pass the start-up period. This paper combines the start-up period and growth period into the growth period; the enterprise life cycle is divided into two stages: growth period and maturity period, and the sample was divided into growth period enterprises and maturity period enterprises and retested.

The results of the grouped regressions are shown in Table 8, where (1) is the regression result of the sample of mature stage enterprises and (2) is the regression result of the sample of mature stage enterprises. The results show that the coefficient of Post*SOE is insignificant in the sample of mature-period firms and the coefficient of Post*SOE is significantly positive at the 5% level in the sample of mature-period firms. This indicates that employee compensation control has a more significant effect on labor productivity improvement in mature-period SOEs, and hypothesis H5 is verified.

**TABLE 8:** Growth stage and maturation stage.

	(1)	(2)
	*Labpro*	*Labpro*
Post*SOE	0.0384	0.1062**
	(0.8359)	(2.1963)
*Size*	0.2385***	0.2175***
	(11.8864)	(11.0381)
*Lev*	0.3353***	0.4368***
	(2.8652)	(3.2137)
*Cash*	0.2251*	0.4023**
	(1.6675)	(2.3840)
*Growth*	0.1896***	0.1717***
	(5.5433)	(5.7613)
*ROE*	0.4991***	0.4903***
	(3.9580)	(3.8018)
*Dual*	−0.1023***	−0.0812*
	(−2.6772)	(−1.8970)
*Conc*	0.0030**	0.0032**
	(2.3652)	(2.3732)
*Direct*	−0.0088	−0.0103
	(−0.7139)	(−0.7993)
*Inde*	−0.2263	−0.6116*
	(−0.6713)	(−1.6520)
*Age*	0.0043	−0.0056
	(0.9795)	(−1.2948)
Constant	8.3775***	8.7912***
	(18.8500)	(19.9739)
Year FE	Yes	Yes
Industry FE	Yes	Yes
Province FE	Yes	Yes
N	4,575	4,892
Adj-R2	0.3503	0.3628

T-values are reported in parentheses; *,** and *** indicate statistical significance at the 1%, 5%, and 10% levels, respectively.

## Conclusion

The policy of employee compensation restriction in state-owned enterprises is an important reform in regulating the national income gap and promoting common prosperity. Whether employee compensation restriction can reconcile the issues of “efficiency” and “equity” and achieve its policy objectives is a question to be explored. This paper examines the direct policy effects of employee compensation restriction from the perspective of labor productivity and aims to provide useful ideas for SOEs to optimize their income distribution system. Using the promulgation of the “Interim Measures on the Management of Central Enterprises’ Total Wage Budget” as a quasi-natural experiment, we empirically examined the mechanism of employee compensation control on labor productivity of SOEs. We found that: (1) Employee compensation restriction enhances the labor productivity of SOEs, and the findings remain unchanged after a series of robustness testing procedures. (2) This policy effect is mainly due to the contribution of employee compensation controls to the external fairness of employee compensation. (3) The effect of employee compensation restriction on labor productivity improvement is more significant in monopolistic industries or mature SOEs.

The findings of this paper support the efficiency-enhancing effect of employee compensation control as an equity-promoting policy on SOEs and also provide micro-level empirical evidence for high-quality economic development. In state-owned capital supervision and management departments, the reform of the wage system for government department employees should be promoted to fully mobilize employee motivation by exploring a mechanism linking the compensation of employees in state-owned enterprises, institutions, and government departments to efficiency and market levels to achieve a balance between fairness and efficiency. In addition, the promotion of employee pay control needs to strengthen policy precision, adhere to the idea of classification and control, differentiate control according to the competitive situation of different industries and the level of demand of employees in enterprises with different life cycles, and enhance the policy effect of employee pay control.

Our study has the following limitations. First, state-owned enterprises have undergone substantial reforms in many areas in recent years. Unlike other national regulations that typically affect all listed firms, employee compensation controls are only implemented in state-listed firms (wholly state-owned versus state-controlled enterprises). This allows us to test our research questions and conduct a series of robustness tests using a DID research design, but we acknowledge that our tests can mitigate but not fully rule out potentially confounding effects. Second, the scope of SASAC controls on employee compensation is also in non-listed firms, and we lack the data to test the impact of employee compensation controls on non-listed SOEs as these firms do not disclose husband and performance data. We acknowledge this limitation and call for future research to shed light on this issue when more data becomes available.

## Data availability statement

The original contributions presented in the study are included in the article/supplementary material, further inquiries can be directed to the corresponding author.

## Author contributions

BZ and ZM: conceptualization and formal analysis. BZ: methodology, software, resources, data curation, writing – original draft preparation, writing, review, and editing. ZM: validation. XQ: investigation. All authors have read and agreed to the published version of the manuscript.

## References

[B1] Adams J. S. (1963). Toward an understanding of inequity. *J. Abnorm. Soc. Psychol.* 67 422–436. 10.1037/h0040968 14081885

[B2] Aivazian V. A. Ying G. Qiu J. (2005). Can corporatization improve the performance of state-owned enterprises even without privatization? *J. Corp. Financ.* 11 791–808. 10.1016/j.jcorpfin.2004.11.001

[B3] Autor D. H. Manning A. Smith C. (2016). The contribution of the minimum wage to US wage inequality over three decades: A reassessment. *Am. Econ. J. Appl. Econ.* 8 58–99. 10.1257/app.20140073

[B4] Babenko I. Lemmon M. Tserlukevich Y. (2011). Employee stock options and investment. *J. Financ.* 66 981–1009. 10.1111/j.1540-6261.2011.01657.x

[B5] Bai M. Wang R. Yu C. Zheng J. (2019). Limits on executive pay and stock price crash risk: Evidence from a quasi-natural experiment. *Pac. Basin Financ. J.* 55 206–221. 10.1016/j.pacfin.2019.04.003

[B6] Bao Q. Ye N. H. Shao M. (2014). Export learning, heterogeneous matching and dynamic changes of firm productivity. *J. World Econ.* 37 26–48.

[B7] Baron R. M. Kenny D. A. (1987). The moderator-mediator variable distinction in social psychological research: Conceptual, strategic, and statistical considerations. *J. Pers. Soc. Psychol.* 51 1173–1182. 10.1037/0022-3514.51.6.1173 3806354

[B8] Beatty A. (1995). The cash flow and informational effects of employee stock ownership plans. *J. Financ. Econ.* 38 211–240. 10.1016/0304-405X(94)00812-F

[B9] Bechtel M. M. Liesch R. Scheve K. F. (2018). Inequality and redistribution behavior in a give-or-take game. *Proc. Natl. Acad. Sci. U.S.A.* 115 3611–3616. 10.1073/pnas.1720457115 29555734 PMC5889654

[B10] Beck T. Levine R. Levkov A. (2010). Big bad banks? The winners and losers from bank deregulation in the United States. *J. Financ.* 65 1637–1667. 10.1111/j.1540-6261.2010.01589.x

[B11] Bender S. Bloom N. Card D. Reenen J. V. Wolter S. (2018). Management practices, workforce selection, and productivity. *J. Labor Econ.* 36 S371–S409. 10.1086/694107

[B12] Brandt L. Li H. (2003). Bank discrimination in transition economies: Ideology, information, or incentives? *J. Comp. Econ.* 31 387–413. 10.1016/S0147-5967(03)00080-5

[B13] Camerer C. F. (2003). Behavioural studies of strategic thinking in games. *Trends Cogn. Sci.* 7 225–231. 10.1016/S1364-6613(03)00094-9 12757825

[B14] Card D. Mas A. Moretti E. Saez E. (2012). Inequality at work: The effect of peer salaries on job satisfaction. *Am. Econ. Rev.* 102 2981–3003. 10.2307/41724678

[B15] Cengiz D. Dube A. Lindner A. Zipperer B. (2019). The effect of minimum wages on low-wage jobs. *Q. J. Econ.* 3 1405–1454. 10.1093/qje/qjz014

[B16] Chen Y. Chen D. Wang W. Zheng D. (2018). Political uncertainty and firms’ information environment: Evidence from China. *J. Account. Public Policy* 37 39–64. 10.1016/j.jaccpubpol.2018.01.005

[B17] Cheng H. (2018). Does management improve labor productivity? *Manag. World* 34 86–99.

[B58] Chung Y. T. (2002). Monopoly, employment and wages. *Labour Econ*. 9, 681–697. 10.1016/S0927-5371(02)00057-X

[B18] Conyon M. J. He L. (2011). Executive compensation and corporate governance in China. *J. Corp. Financ.* 17 1158–1175. 10.1016/j.jcorpfin.2011.04.006

[B19] Dickinson V. (2011). Cash flow patterns as a proxy for firm life cycle. *Account. Rev.* 86 1969–1994. 10.2308/accr-10130

[B20] Dittmann I. Maug E. Zhang D. (2011). Restricting CEO pay. *J. Corp. Financ.* 17 1200–1220. 10.1016/j.jcorpfin.2011.04.007

[B21] Dube A. Lester T. W. Reich M. (2010). Minimum wage effects across state borders: Estimates using contiguous counties. *Rev. Econ. Stat.* 92 945–964. 10.1162/REST_a_00039

[B22] Ellig B. R. (1981). Compensation elements: Market phase determines the mix. *Compensation Benefits Rev. J. Total Compensation Strat.* 13 30–38. 10.1177/088636878101300303

[B23] Fang H. Nofsinger J. R. Quan J. (2015). The effects of employee stock option plans on operating performance in Chinese firms. *J. Bank. Financ.* 54 141–159. 10.1016/j.jbankfin.2015.01.010

[B24] Fehr E. Gächter S. (2002). Altruistic punishment in humans. *Nature* 415 137–140. 11805825 10.1038/415137a

[B25] Gan L. Hernandez M. A. Ma S. (2016). The higher costs of doing business in China: Minimum wages and firms’ export behavior. *J. Int. Econ.* 100 81–94. 10.1016/j.jinteco.2016.02.007

[B26] Gao X. Yu H. Ignacio S. Blue P. R. Zhu L. Ming H. (2018). Distinguishing neural correlates of context-dependent advantageous- and disadvantageous-inequity aversion. *Proc. Natl. Acad. Sci. U.S.A.* 415 E7680–E7689. 10.1073/pnas.1802523115 30061413 PMC6099874

[B27] Gordon R. H. Wei L. (2003). Government as a discriminating monopolist in the financial market: The case of China. *J. Public Econ.* 87 283–312. 10.1016/S0047-2727(01)00144-X

[B28] Hannan R. L. (2005). The combined effect of wages and firm profit on employee effort. *Account. Rev.* 80 167–188. 10.2308/accr.2005.80.1.167

[B29] Haveman H. A. Jia N. Shi J. Wang Y. (2017). The dynamics of political embeddedness in China. *Adm. Sci. Q.* 62 67–104. 10.1177/0001839216657311

[B30] Hilscher J. Landskroner Y. Raviv A. (2021). Optimal regulation, executive compensation and risk taking by financial institutions. *J. Corp. Financ.* 71:102104. 10.1016/j.jcorpfin.2021.102104

[B31] Hoang N. Nahm D. Dobbie M. (2021). Innovation, gender, and labour productivity: Small and medium enterprises in Vietnam. *World Dev.* 146:105619. 10.1016/j.worlddev.2021.105619

[B32] Holzer H. J. (1990). Wages, employer costs, and employee performance in the firm. *ILR Rev.* 43 147S–164S. 10.1177/001979399004300310

[B33] Ibrahim S. Li H. Yan Y. Zhao J. (2021). Pay me a single figure! Assessing the impact of single figure regulation on CEO pay. *Int. Rev. Financ. Anal.* 73:101647. 10.1016/j.irfa.2020.101647

[B34] Jian J. Li H. Meng L. Zhao C. (2020). Do policy burdens induce excessive managerial perks? Evidence from China’s stated-owned enterprises. *Econ. Model.* 90 54–65. 10.1016/j.econmod.2020.05.002 32372842 PMC7199001

[B35] Kale J. R. Ryan H. E. Wang L. (2019). Outside employment opportunities, employee productivity, and debt discipline. *J. Corp. Financ.* 59 142–161. 10.1016/j.jcorpfin.2016.08.005

[B36] Kim T. Y. Leung K. (2007). Forming and reacting to overall fairness: A cross-cultural comparison. *Organ. Behav. Hum. Decis. Process.* 104 83–95. 10.1016/j.obhdp.2007.01.004

[B37] Klenow H. P. J. (2009). Misallocation and manufacturing TFP in China and India. *Q. J. Econ.* 124 1403–1448.

[B38] Kong D. Wang Y. Zhang J. (2020). Efficiency wages as gift exchange: Evidence from corporate innovation in China. *J. Corp. Financ.* 65:101725. 10.1016/j.jcorpfin.2020.101725

[B39] Leonard J. S. (1987). Carrots and sticks: Pay, supervision, and turnover. *J. Labor Econ.* 5 S136–S152.

[B40] Li H. Meng L. Qian W. Zhou L. A. (2008). Political connections, financing and firm performance: Evidence from Chinese private firms. *J. Dev. Econ.* 87 283–299. 10.1016/j.jdeveco.2007.03.001

[B41] Li J. Miao E. Zhang J. (2021). The legal environment, specialized investments, incomplete contracts, and labor productivity. *China Econ. Rev.* 66:101583. 10.1016/j.chieco.2021.101583

[B42] Li O. Xu F. Wang L. (2018). Advantageous inequity aversion does not always exist: The role of determining allocations modulates preferences for advantageous inequity. *Front. Psychol.* 9:749. 10.3389/fpsyg.2018.00749 29887817 PMC5981801

[B43] Li P. Lu Y. Wang J. (2016). Does flattening government improve economic performance? Evidence from China. *J. Dev. Econ.* 123 18–37. 10.1016/j.jdeveco.2016.07.002

[B44] Liao L. Liu B. Wang H. (2014). China’s secondary privatization: Perspectives from the split-share structure reform. *J. Financ. Econ.* 113 500–518. 10.1016/J.JFINECO.2014.05.007

[B45] Peng J. Xie R. Ma C. Fu Y. (2021). Market-based environmental regulation and total factor productivity: Evidence from Chinese enterprises. *Econ. Model.* 95 394–407. 10.1016/j.econmod.2020.03.006

[B46] Pillai R. Williams E. S. Tan J. J. (2001). Are the scales tipped in favor of procedural or distributive justice? An investigation of the U.S., India, Germany, and Hong Kong (China). *Int. J. Confl. Manag.* 12 312–332. 10.1108/eb022861

[B47] Rousseau P. L. Xiao S. (2008). Change of control and the success of China’s share-issue privatization. *China Econ. Rev.* 19 605–613. 10.1016/j.chieco.2008.06.003

[B48] Schultz T. W. (1961). Investment in human capital. *Am. Econ. Rev.* 51 1–17.

[B49] Shapiro C. Stiglitz J. E. (1984). Equilibrium unemployment as a worker discipline device. *Am. Econ. Rev.* 74 433–444.

[B50] Wang Y. (2014). SOE payroll budget management to deepen and expand. *Hum. Resour. Dev. China.* 14:38–42. 10.16471/j.cnki.11-2822/c.2014.14.014

[B51] Warner M. (2010). Management-labour relations in the new Chinese economy. *Hum. Resour. Manag. J.* 7 30–43. 10.1111/j.1748-8583.1997.tb00287.x

[B52] Xi J. K. Moser D. V. (2011). Wage negotiation, employee effort, and firm profit under output-based versus fixed-wage incentive contracts*. *Contemp. Account. Res.* 28 616–642. 10.1111/j.1911-3846.2010.01050.x

[B53] Yu R. Calder A. J. Mobbs D. (2013). Overlapping and distinct representations of advantageous and disadvantageous inequality. *Hum. Brain Mapp.* 35 3290–3301. 10.1002/hbm.22402 25050425 PMC4216415

[B54] Yue G. (1985). Employment, wages and social security in China. *Int. Labour Rev.* 124 411–422.

[B55] Zhang H. Jianyu Z. Zhengfei L. (2021). Employee salary competitiveness and the adoption of employee stock ownership plan. *J. Financ. Res.* 487 169–187.

[B56] Zhou H. (2004). High benefits and low wages: Employees as monitor of management in SOEs. *China Econ. Rev.* 15 407–423. 10.1016/j.chieco.2004.06.006

[B57] Zhu C. J. Dowling C. (1998). Cross-cultural and comparative international human resource management: The reform of employee compensation in China’s industrial enterprises. *Mir Manag. Int. Rev.* 38 65–87. 10.2307/40228479

